# School Children

**Published:** 1910-07-30

**Authors:** 


### SCHOOL CHILDREN.

### SOME REFLECTIONS ON PRESENT ARRANGEMENTS.

As the arrangements which were made for twelve months between the London County Council and the London hospitals for the treatment of school children are about to come to an end, it is appropriate to consider this important question and the way it may affect medical men. In the first place, two broad principles may be laid down. The first of these concerns the working of the Children Act. This Act made compulsory the medical inspection of school children, and gave education authorities the option of treating such children as are found defective. It is quite obvious that inspection without treatment is almost valueless, and could only be done at a cost which would be quite disproportionate to any value that could be got from it. Further, if treatment be paid for out of the rates it is useless to go half-way, and it should be thoroughly and efficiently carried out. Anyone who may have strongly opposed the principle of rate-paid treatment has a perfect right to urge that it be thoroughly done when once it has been introduced.

The second principle that we wish to lay down concerns the medical profession, and it is that if the State puts the burden of treating school children upon the rates, it should not put the further burden on the medical profession of treating such children without payment. That is to say, for every school child treated at the instance of the education authority adequate payment should be given to the medical man who treats that child. In laying down this principle we do not wish to urge practitioners into "the Government stroke." It should not, however, be hard to determine what is a reasonable price at which these children may be treated, and that price should be paid to the man who does the work.

During the past year certain of the London hospitals have made an arrangement with the County Council whereby they have received money and have guaranteed to treat a certain number of the Council's children; we believe, however, that we are correct in saying that little of that money has gone to the medical staff of the hospital, but nearly all towards maintenance. Other hospitals have treated the children in the same way as any other patient coming to their doors, without any payment.

The last method violates both the principles laid down above. The first objection—that the present method is inadequate to the needs of the schools—will no doubt soon be rectified. It has now been proved that the number of children who need treatment is far greater than was ever anticipated, and arrangements cannot be evolved without time for treating all these children.

The second question is more serious. It seems that those in charge of these hospitals have done their medical staffs great wrong in imposing a State work upon them without adequate payment, and the second class are more to be blamed than the first in that they are also using the money given for charitable ends for purposes which the Legislature has laid on the educational authorities, and for which the State should pay. It may be that the charitable public is willing that this money should be so used, but it is doubtful whether a man who has already paid his rates would feel very willing to write a cheque for a hospital if he knew that it would be used for purposes the expenses of which have to be defrayed from the rates.

It is not necessary to argue that the London County Council is doing wrong in sending children to the hospitals. This body has an equal right to get its work done in the cheapest way so long as it is being done efficiently, and so long as it is harming no one by so doing. Those hospitals which are receiving money from the Council for treating these children pay a proportion of that money to its medical staff for such work as the members of the staff may do for the State. But no hospital should undertake to treat any of these children sent by the Council unless it receives a sum from the Council which shall cover the cost of maintenance to the out-patient or in-patient department which that patient represents, and should also provide an adequate remuneration to the professional man who is treating that case. Practitioners who are not attached to a hospital staff have little idea of the extent to which out-patient work has been increased by the attendance of these children, many of whom would never have attended had it not been for the school doctor's warning to the parents. In some children's hospitals the increase probably exceeds 60 per cent. of the total number of new patients daily. It is satisfactory to note that certain institutions have recognised this added burden of work thrown on the out-patient assistant physicians and surgeons, and have appointed paid clinical assistants or have given a certain percentage of their grant to the honorary out-patient officers in consideration for this extra work. This is as it should be, and it is to be hoped that the example set by these hospitals—among which may be mentioned St. George's and the Victoria Hospital for Children, Chelsea—will be followed by all the other institutions which receive London County Council grants for the medical treatment of school children.

